# Characterization of Brachycephalic Obstructive Airway Syndrome in Cats Using Barometric Whole-Body Plethysmography

**DOI:** 10.3390/ani16060959

**Published:** 2026-03-19

**Authors:** Chi-Ru Chen, Alicia Caro-Vadillo, José Alberto Montoya-Alonso, Wei-Tao Chang, Chung-Hui Lin, Laín García-Guasch

**Affiliations:** 1 Lab of Small Animal Respiratory and Cardiovascular Medicine, TACS-Alliance Research Center, Taipei 110, Taiwan; roo201061@gmail.com (C.-R.C.); joey607860@gmail.com (W.-T.C.); joan.chlin@gmail.com (C.-H.L.); 2 National Taiwan University Veterinary Hospital, National Taiwan University, Taipei 106, Taiwan; 3 Internal Medicine & Animal Surgery, Faculty of Veterinary Medicine, Universidad Complutense de Madrid, 28040 Madrid, Spain; aliciac@ucm.es; 4 Internal Medicine & Research Institute of Biomedical and Health Sciences, Universidad de Las Palmas de Gran Canaria, 35413 Las Palmas de Gran Canaria, Spain; alberto.montoya@ulpgc.es; 5 Animal Resource Center and Graduate Institute of Veterinary Clinical Sciences, National Taiwan University, Taipei 106, Taiwan; 6 IVC Evidensia Hospital Veterinari Molins, 08620 Barcelona, Spain; 7 IVC Evidensia Hospital Veterinaria del Mar, 08005 Barcelona, Spain

**Keywords:** barometric whole-body plethysmography, brachycephalic obstructive airway syndrome, cat, pulmonary function test, upper airway obstruction

## Abstract

***Objectives***: To confirm the utility of barometric whole-body plethysmography (BWBP) as a non-invasive, clinical diagnostic test for brachycephalic obstructive airway syndrome (BOAS) in cats. ***Methods***: Client-owned cats belonging to brachycephalic breeds were enrolled and classified into two clinical severity grades of upper airway obstruction (UAO). Brachycephalic cats with high-grade UAO severity (Brachy-H-UAO) represented those with clinically evident effects on clinical signs or physical examination findings, whereas brachycephalic cats with low-grade UAO severity (Brachy-L-UAO) represented those without clinically evident problems. A group of non-brachycephalic (NB) cats that were respiratory disease-free and with neither a history of cardiac or systemic diseases nor exposure to cigarette smoke was used as the control group. Cats were placed in the BWBP chamber, and breathing signals were obtained after an adaptation period in a quiet and silent environment. The ventilatory variables obtained were respiratory rate (RR; [bpm]), tidal and minute volume per kilogram bodyweight (MV/BW and TV/BW; [mL/kg]), inspiratory (Ti; [s]) and expiratory (Te; [s]) intervals, airway obstruction index enhanced pause (Penh), and peak inspiratory and expiratory flows per kilogram (PIF and PEF; [mL/s/kg]). ***Results***: Forty-three client-owned cats (11 Brachy-H-UAO, 7 Brachy-L-UAO, and 25 NB) were included. Brachycephalic cats (Brachy-H-UAO: 311 mL/kg; Brachy-L-UAO: 253 mL/kg) showed significantly lower median MV/BW than NB cats (503 mL/kg) (*p* = 0.01). Brachy-H-UAO cats demonstrated significantly higher median PEF/PIF ratios (Brachy-H-UAO: 1.46, minimum–maximum 0.82–2.48; Brachy-L-UAO: 0.76, 0.52–1.11; NB: 0.73, 0.56–1.00) and Penh (Brachy-H-UAO: 2.37, minimum–maximum 0.57–23.82; Brachy-L-UAO: 0.57, 0.27–1.11; NB: 0.53, 0.21–0.68) than Brachy-L-UAO and NB cats (*p* < 0.001). No significant differences were observed among the three groups for RR, TV/BW, Ti, Te, or Te/Ti. ***Conclusions and Relevance***: Cats affected by BOAS demonstrate impaired ventilatory function, with reduced minute ventilation and a distinctive flow pattern and parameters reflecting limited inspiratory flow and increased upper airway resistance. BWBP can serve as a useful tool to diagnose and characterize the severity of BOAS in cats.

## Simple Summary

Brachycephalic cat breeds refer to cats with a short-nosed or flat-faced conformation, which can be associated with variable severity of upper airway obstruction (UAO) known as brachycephalic obstructive airway syndrome (BOAS). These structural abnormalities may restrict airflow through the upper airway and reduce effective ventilation. This study evaluated whether barometric whole-body plethysmography (BWBP), a non-invasive test that records breathing signals while a cat rests in a chamber after an adaptation period in a quiet environment, can be used clinically to identify and grade UAO severity in brachycephalic cats. Forty-three client-owned cats were enrolled, classified as having high-grade UAO (clinically evident effects on clinical signs or physical examination findings) or low-grade UAO (no clinically evident problems), and compared with healthy non-brachycephalic control cats. Both brachycephalic groups had lower minute ventilation than non-brachycephalic controls, suggesting impaired ventilation. Cats with high-grade UAO also showed additional evidence of limited inspiratory flow and increased upper airway resistance. These results suggest that brachycephalic conformation in cats is associated with functional impairment of ventilation. This study demonstrates that brachycephalic conformation has physiological impacts and should be recognized as a welfare concern in cats. Additionally, BWBP’s ability to detect these changes highlights its value as a diagnostic and grading tool for assessing UAO in cats.

## 1. Introduction

Brachycephalic obstructive airway syndrome (BOAS) arises from craniofacial shortening and the resulting upper airway obstruction (UAO) [1,2,3]. In cats, particularly Persians and Exotic Shorthairs, extreme brachycephaly is characterized by structural abnormalities such as stenotic nares, elongated soft palate, compressed nasal turbinates, narrowed nasal apertures, and nasolacrimal duct distortion, dental malalignment, and exophthalmos [2,3,4,5,6,7,8]. These conformational changes increase airway resistance and are associated with breath noise, respiratory distress, exercise intolerance, nasal discharge, and ocular problems [1,2,3,6,7,8]. Population-based studies and owner surveys have consistently reported higher prevalence of respiratory, ocular, and dental disorders in brachycephalic cats, with more frequent snoring, sneezing, and reduced activity levels [1,3,6]. Shorter facial conformation was associated with higher owner-reported respiratory noise and/or breathing difficulty via a composite respiratory score [1]. Owner questionnaire data also indicate that brachycephalic cats commonly exhibit respiratory signs, with snoring and sneezing reported more than 1–2 times/week in 53–55% and activity-associated dyspnea in 31% of brachycephalic cats (vs. 14–18% and 9% in non-brachycephalic cats, respectively) [6].

While UAO represents a key feature of feline BOAS, objective methods for evaluating upper airway airflow limitations are still lacking in feline medicine. Stertor and snoring are commonly noted but remain difficult to quantify, and their assessment is often compromised by subjectivity among clinicians [4]. Reliance on owner-reported questionnaires to assess UAO severity is also limited by perceptual bias, as some owners may not recognize mild abnormalities, potentially leading to underestimation of disease severity [3]. Imaging modalities such as computed tomography can provide valuable structural information [2,4]; however, the physiological consequences and functional impact of UAO on ventilation cannot be evaluated through structural imaging alone.

Respiratory or pulmonary function testing is routinely used in human respiratory medicine. Nevertheless, cats are unable to cooperate with forced maneuvers that require maximal voluntary effort when conscious, limiting direct translation of standard human spirometry protocols to feline patients. Although direct measurements of lung compliance and airway resistance can provide important insights into ventilatory mechanics and have been performed in research cats and selected clinical cases, these procedures typically require general anesthesia and endotracheal intubation, which is often not acceptable for many pet owners owing to increased risk for respiratory patients. Barometric whole-body plethysmography (BWBP) is a non-invasive pulmonary function test that enables dynamic assessment of breathing patterns in unrestrained and unsedated cats [9,10,11]. BWBP was performed in awake, unrestrained cats in a quiet setting. It enables an animal to stay and move freely within the test chamber, minimizing stress during the evaluation, avoiding sedation/anesthesia and minimizing handling stress while providing objective functional respiratory measurements. Owing to its cat-friendly nature, BWBP has been increasingly applied in clinical feline respiratory medicine across a wide range of conditions, including feline lower airway disease, obesity-related respiratory compromise, parasitic infections, and cats with labored breathing [12,13,14,15,16,17,18,19,20,21,22,23]. To address the absence of a quantitative assessment tool for feline UAO, previous attempts have explored the use of BWBP in cats with nasal neoplasia, stenotic nares, nasopharyngeal stenosis, tracheal mass, and laryngeal mass [24,25]. Although BWBP has been employed to investigate canine BOAS [26,27,28], the ventilatory effects in brachycephalic cats remain underinvestigated. Therefore, the aim of this study was to confirm the utility of BWBP as a clinical diagnostic test for BOAS in cats.

## 2. Materials and Methods

### 2.1. Study Population

Client-owned cats belonging to brachycephalic breeds were retrospectively or prospectively enrolled in two institutions. Brachycephalic cats were classified into two clinical severity grades primarily based on UAO-associated clinical signs and physical examination findings; the owner’s perspective was considered supportive information (Table 1). The high-grade UAO group (Brachy-H-UAO) represented cats with clinically evident UAO-associated abnormalities on history or physical examination, whereas the low-grade UAO group (Brachy-L-UAO) represented cats without clinically evident UAO-associated findings. Non-brachycephalic cats (NB cats) were either prospectively enrolled from health examinations or retrospectively included from historical control groups of previous functional studies, and none of the cats had a history of cardiac or systemic diseases or exposure to cigarette smoke. A priori sample size calculation was not performed because no published data on the use of BWBP in assessing brachycephalic cats with graded upper airway obstruction were available to support a reliable effect size estimate at study conception. Therefore, all eligible client-owned brachycephalic cats meeting the inclusion criteria during the study period at the participating institutions were included. While ethical approval was not mandatory in all participating institutions due to the non-invasive nature of BWBP and the known clinical usefulness of the technique, informed consent was consistently obtained from all cat owners prior to participation across all study sites. This study is reported in accordance with the ARRIVE guidelines (Supplementary Materials).

Forty-three client-owned cats were enrolled, including 11 Brachy-H-UAO cats, 7 Brachy-L-UAO cats, and 25 NB cats. Overall, the breeds included Domestic Shorthair (25), Persian (10), British Shorthair (3), Himalayan (2), and one each of Scottish Fold, Siamese, and Russian Blue. Brachy-H-UAO group consisted of Persian (6), DSH (2) and one each of British Shorthair, Himalayan, and Scottish Fold. The Brachy-L-UAO group consisted of Persian (4), British Shorthair (2), and Himalayan (1). The NB group consisted of DSH (23) and one each of Siamese and Russian Blue.

### 2.2. Barometric Whole-Body Plethysmography

**Figure 1:**
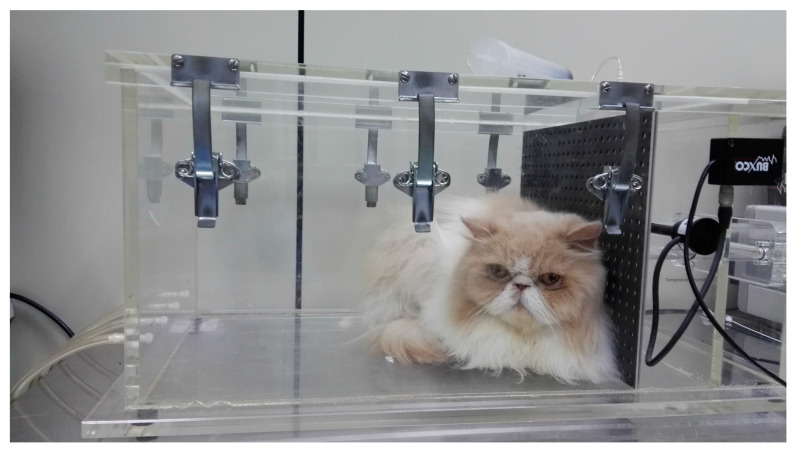
A brachycephalic cat calmly resting in the BWBP chamber in a quiet room. The cat was awake and unrestrained during the whole assessment, demonstrating the cat-friendly nature of BWBP.

BWBP was performed using the same method previously described [13,15,16,17]. The system was calibrated before each test by injecting 50 mL of air with a calibrated syringe according to the manufacturer’s instructions. Cats were then placed without restraint in a transparent Plexiglas chamber in a quiet room. An acclimation period of several minutes was allowed until the cat appeared settled, as indicated by a comfortable posture and stable breathing signals, before recording (Figure 1). When a cat breathes in the chamber, two components contribute to the measured signal: nasal airflow at the nares and respiratory thoracic displacement. Because inspired air is warmed and humidified within the airways, the apparent volume change reflected by thoracic displacement is greater than the volume change represented by nasal airflow at the nares. The net difference between these components drives gas to move into or out of the chamber, and this “box flow” is measured as flow through the pneumotach mounted on the chamber wall via the pressure drop across its resistive metal mesh. Therefore, the signal detected at the pneumotach is not solely nasal airflow but the net result of these two components and is commonly referred to as “pseudoflow” or “box flow.” The pseudoflow and pseudo-volume differ from, but correspond to, the animal’s true flow and volume [22]. A bias flow of 6–10 L/min of room air was provided throughout the examination. Signals were acquired with a differential pressure transducer, amplified, digitized, and analyzed with BioSystem XA software (v2.10.1 or v2.11.0, Buxco Electronics, Wilmington, NC, USA). Each recording lasted 10 to 12 min, during which room-air breathing was measured.

Ventilatory variables derived included respiratory rate (RR, breaths/min), tidal volume (TV, mL; TV/BW, mL/kg), minute volume (MV, mL; MV/BW, mL/kg), inspiratory and expiratory times (Ti, Te, s), peak inspiratory and expiratory flows (PIF, PEF, mL/s or mL/s/kg), relaxation time (RT, s, time at which 65% of tidal volume is exhaled), pause (PAU, unitless, (Te − RT)/RT) and enhanced pause (Penh, unitless, (PEF/PIF) × (Te − RT)/RT). Non-respiratory or artifactual waveforms (e.g., body movement, vocalization, sniffing, and posture changes) were excluded by computed rejection settings or manual deletion. BWBP parameters were averaged across accepted breaths and directly exported to Microsoft Excel for subsequent analysis.

### 2.3. Statistical Methods

Statistical analyses were conducted using commercially available software (SPSS version 27, IBM Corp, Armonk, NY, USA). The Shapiro–Wilk test was applied to evaluate the normality of continuous variables. Data with a normal distribution are expressed as mean ± SD, whereas non-normally distributed data are expressed as median and IQR. Baseline signalment variables among Brachy-H-UAO, Brachy-L-UAO, and NB cats were compared using one-way ANOVA, Mann–Whitney U-test, or Chi-square test, as appropriate. BWBP ventilatory variables were compared using the Kruskal–Wallis test followed by post hoc analyses with Bonferroni correction, when indicated. For Kruskal–Wallis tests, an effect size (eta-squared based on the H statistic; η^2^H) was calculated to quantify the magnitude of between-group differences. Negative values were set to 0. For all analyses, a *p*-value < 0.05 was considered statistically significant.

## 3. Results

Of the study population, 55.8% (24/43) were female. Baseline characteristics were not different between groups (Table 2).

**Figure 2:**
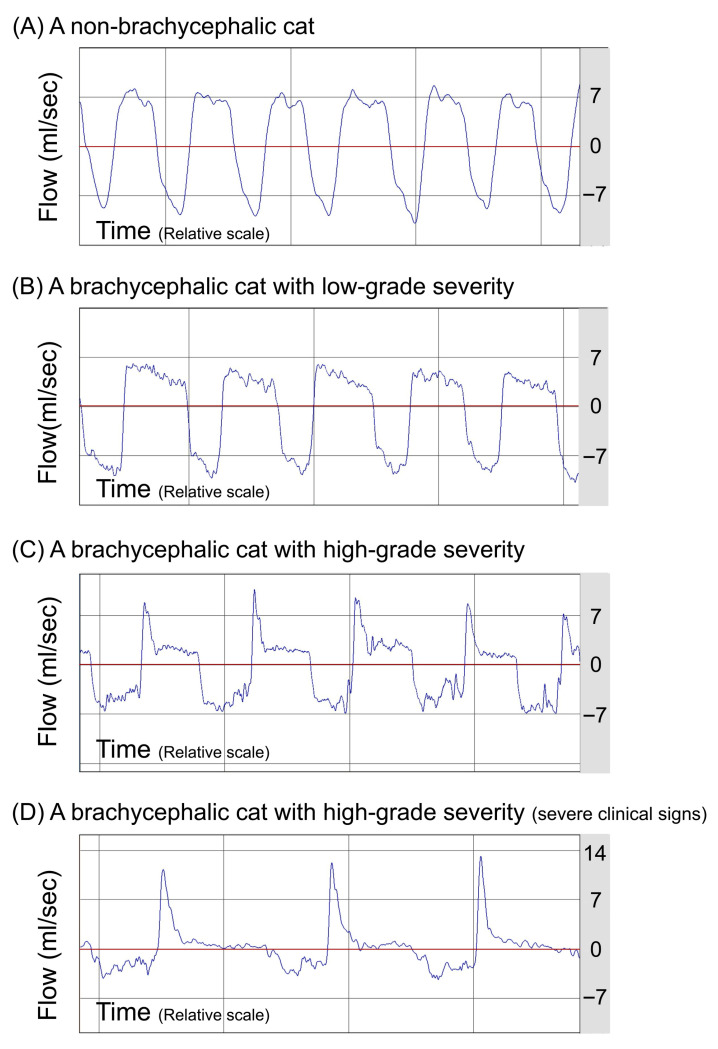
Representative BWBP pseudoflow signals from cats without and with varying severities of brachycephaly and airflow limitation. The horizontal red line indicates the zero-flow baseline. Upward deflections represent expiratory flow, and downward deflections represent inspiratory flow. (**A**) Non-brachycephalic (NB) cat showing a stable and normal waveform. (**B**) Brachycephalic cat with low-grade upper airway obstruction (Brachy-L-UAO) showing relatively preserved waveforms similar to NB. (**C**,**D**) Brachycephalic cats with high-grade upper airway obstruction (Brachy-H-UAO) demonstrating patterns consistent with inspiratory airflow limitation, with disproportionately reduced peak inspiratory flow relative to peak expiratory flow, most pronounced in (**D**), a clinically severe case.

The ventilatory parameters of BWBP were compared between groups. No differences were found for RR, TV/BW, Te, Ti, and the Te/Ti ratio between groups. However, significant differences in BWBP ventilatory parameters between groups were identified for MV/BW, PIF/BW, PEF/BW, PEF/PIF, PAU, and Penh (Table 3). Both Brachy-H-UAO (*p* = 0.03) and Brachy-L-UAO cats (*p* = 0.04) exhibited significantly lower MV/BW compared to NB cats, regardless of their clinical status. PEF/PIF and Penh were significantly higher in Brachy-H-UAO cats compared with the other groups (Table 3). Brachy-H-UAO cats exhibited clear airflow obstruction on pseudoflow tracings, whereas NB cats showed normal pseudoflow tracings without evidence of airflow limitation (Figure 2).

## 4. Discussion

In this study, brachycephalic conformation was associated with measurable ventilatory impairment, with the greatest functional abnormalities observed in clinically affected cats. Brachycephalic cats had significantly lower MV than non-brachycephalic controls. In contrast, indices more consistent with airflow limitation, particularly an increased PEF/PIF ratio and elevated Penh, were significantly higher only in cats with clinically evident UAO, supporting greater inspiratory flow limitation and altered breathing mechanics in this subgroup. These results support the clinical utility of BWBP to objectively quantify ventilatory compromise in feline BOAS and to help distinguish clinically affected from clinically unaffected brachycephalic cats when interpreted alongside history and physical examination.

Brachycephalic conformation is associated with a variable combination of stenotic nares, elongated soft palate, abnormal nasal turbinates, and other abnormalities, and all these abnormalities contribute to UAO to a variable extent [2,3,4,7,8]. In our study, lower MV observed in brachycephalic cats compared to those with normal head conformation implies a state of hypoventilation, which has been recognized as one of the possible consequences of chronic UAO [29,30]. Notably, this implication should be interpreted with caution because the measured minute volume includes ventilation of the conducting airways and therefore does not represent alveolar ventilation. In contrast, studies in dogs did not detect a significant difference in MV between brachycephalic and non-brachycephalic breeds [26,27]. However, cats with UAO seem to exhibit a different ventilatory feature compared to dogs. A majority of cats with labored breathing showed elevated MV in a previous study using BWBP assessment, but cats with UAO demonstrated a different trend when compared to those with lower airway obstruction, lung parenchymal disease, or pleural space disorders [22]. Although that study included only five cats with UAO due to other etiologies such as nasopharyngeal stenosis and dynamic nasopharyngeal collapse, and none were related to brachycephalic syndrome, the findings nonetheless suggested a distinct ventilatory response in feline UAO compared to other respiratory pathologies. When severe pathologies compromise the gas exchange capacity, higher MV is required to remove the accumulated carbon dioxide. This increase in MV is a compensatory response, aiming to maintain sufficient oxygenation and ensure adequate removal of carbon dioxide, thus balancing the increased physiological dead space or impaired gas exchange caused by the pathologies [22]. Cats presented with labored breathing due to UAO etiologies did not reveal an increased in MV, despite the conspicuous respiratory distress, which may indicate a limited capacity to augment ventilation in the setting of upper airway flow limitation in these cats. Our current findings support that brachycephaly in cats leads to impaired minute ventilation, reflecting one of the consequences of long-term ventilatory compromise. It is not known whether cats with profoundly decreased MV might be at higher risk of developing pulmonary hypertension or cor pulmonale as observed in humans [29,31], but this warrants future investigation.

In the present study, the largest effect among all the evaluated ventilatory variables was observed for PEF/PIF, supporting that variables reflecting inspiratory flow limitation and airflow obstruction show the most robust discrimination between cats with clinically evident UAO and those without. Clinically affected brachycephalic cats showed increased PEF/PIF, consistent with the classic pattern of dynamic extrathoracic obstruction described in the previous studies [24,26,32,33,34,35]. The change is mainly due to a predominant restriction of inspiratory flow, with expiratory flow comparatively preserved, thereby raising the expiratory-to-inspiratory flow ratios. In dynamic extrathoracic obstruction, the negative pressure gradient created during inspiration tends to collapse the narrowed airway. Conversely, during forced expiration, the positive pressure within the trachea tends to hold the lesion open, resulting in a relatively normal expiratory flow [32,33,35]. Supporting this interpretation, dogs with brachycephalic syndrome assessed by BWBP exhibited higher PEF/PIF and Penh than controls, with PEF/PIF decreasing after corrective surgery [26]. Such changes have been attributed primarily to extrathoracic upper-airway obstruction. Similarly, in dogs with extrathoracic tracheal collapse, PEF/PIF increases and correlates with disease severity [34]. Moreover, a pilot study in cats with UAO reported significantly higher PEF/PIF than in normal cats, indicating inspiratory airflow limitation [24]. In our study, while both groups of brachycephalic cats revealed decreased MV compared to NB cats, only clinically affected brachycephalic cats demonstrated a consistently higher PEF/PIF ratio, suggesting PEF/PIF may be a more suitable BWBP ventilatory variable in discriminating cats with and without clinically evident inspiratory airflow limitation. Taken together, these observations align with our finding that Brachy-H-UAO cats, compared with Brachy-L-UAO and NB cats, demonstrated a higher PEF/PIF ratio, a pattern physiologically consistent with more severe inspiratory airflow limitation and increased upper-airway resistance in clinically affected BOAS cats.

In the present study, clinically affected brachycephalic cats exhibited significantly elevated Penh values, as anticipated. The Penh index was originally introduced as a surrogate marker of bronchoconstriction in rodent experimental models, and later applied to cats and dogs [9,10,11,36]. It was subsequently reported to be elevated in brachycephalic dogs both before and after surgery compared to healthy controls [26]. Notably, in that study, surgical intervention did not result in a significant change in Penh values; however, the PEF/PIF ratio significantly decreased postoperatively, better reflecting the surgical effect [26]. These findings suggest that different ventilatory indices may serve distinct purposes and should be selected accordingly to ensure appropriate interpretation in clinical pulmonary function testing. Moreover, it is important to recognize that Penh is not a specific indicator of UAO, as it is known to increase in association with lower airway obstruction and various pulmonary conditions [13,14,16,18,20]. This nonspecificity stems from the fact that Penh is not a direct measurement, but rather a complicated calculation derived from multiple variables ([PEF/PIF] × [Te-RT]/RT), including expiratory time, relaxation time (time at which 65% of tidal volume is exhaled), PIF, and PEF [37,38]. Because Penh is influenced by several variables rather than a single physiological measurement, its clinical interpretation is not always intuitive, and should not be simply interpreted as a marker of bronchoconstriction or an indicator of UAO. Given its composite and indirect nature, elevated Penh values should be interpreted with caution, and the inherent limitations and ongoing controversies regarding its clinical relevance must be carefully acknowledged [37,38].

The five domains of animal welfare state that animals should be free from disease, discomfort and able to exhibit normal behaviors [39]. Brachycephaly, defined as an “intentionally bred hereditary disease” [40], constitutes a direct violation of these principles. In dogs, brachycephaly is linked to respiratory distress, aspiration pneumonia, exercise intolerance, syncope, impaired thermoregulation, as well as complications involving other organ systems [26,27,40,41]. Similarly, studies in brachycephalic cats have reported associations with respiratory distress as well as gastrointestinal, dermatological, ophthalmological, and dental disorders [1,2,6]. Consistent with these reports, similar respiratory function compromise was recognized in cats in this study, with brachycephalic individuals exhibiting reduced minute ventilation, inspiratory airflow limitation, and increased upper airway resistance under BWBP assessment. These observations indicate that brachycephaly is not exclusive to dogs but represents a cross-species welfare concern in cats. While diagnostic refinement is valuable, the primary objective should always be the prevention of such phenotypes through discouragement of breeding affected conformations and improving education of veterinarians, breeders, and the general public regarding the health consequences of brachycephaly.

This study has several limitations. First, not all cats underwent a comprehensive evaluation of the upper airway structures by endoscopy or computed tomography to confirm the presence and nature of the primary and secondary components of BOAS and to rule out other potential causes of UAO that are distinct from BOAS. This was due to ethical considerations, as there is currently no evidence supporting the long-term benefits of corrective surgery for feline brachycephaly. Consequently, it is generally not reasonable for owners to pursue further diagnostics requiring anesthesia, except in the case of stenotic nares, which can be assessed without sedation. Therefore, it remains possible that other upper airway disorders contributed to the BWBP waveforms and derived metrics that were attributed to BOAS in this study. Second, although no findings from the history, physical examination, or available medical records suggested concurrent lower airway disease, advanced lower airway assessment was not performed, which limits the interpretation of BWBP findings and makes it difficult to attribute the observed waveform and ventilatory changes to brachycephaly alone. Advanced diagnostics that could more definitively exclude subclinical lower airway disease (e.g., thoracic CT, bronchoalveolar lavage, or tracheal wash) were not performed because they require general anesthesia and were not ethically or practically justified solely for study purposes in otherwise stable client-owned cats. Therefore, subclinical lower airway disease cannot be completely ruled out. While increases in indices such as PEF/PIF and Penh may be compatible with dynamic UAO, some patterns could also be consistent with concurrent dynamic lower airway obstruction. Third, unlike dogs, cats are not typically subjected to high levels of exercise. Because exercise endurance cannot be objectively evaluated, it is possible that some cats classified as clinically unaffected may have had unrecognized clinical impairment. Fourth, as our study was designed around a non-invasive functional assessment in awake client-owned cats, complementary measures of oxygenation and ventilation, such as arterial blood gas analysis, were not performed. Future studies combining arterial blood gas analysis with BWBP could help to better interpret the clinical relevance of the observed ventilatory indices and airflow patterns. Finally, the relatively small sample size, particularly in the Brachy-L-UAO group, may have limited power to detect small between-group differences. In addition, multiple BWBP endpoints were evaluated; therefore, findings should be interpreted in the context of effect sizes and physiologic plausibility.

## 5. Conclusions

Brachycephalic conformation in cats is associated with functional impairment of ventilation, even in those not yet exhibiting significant clinical signs. This study demonstrates that brachycephalic conformation has physiological impacts and should be recognized as a welfare concern in cats. While BWBP requires specialized equipment and expertise in implementation and interpretation, it provides a tool for non-invasive and objective assessment of ventilatory function and airflow limitation method to characterize ventilatory function and airflow limitation and may serve as a complementary tool to support clinical assessment and grading of UAO in cats.

## Figures and Tables

**Table 1:** Definitions used to classify the two clinical severity grades of upper airway obstruction in brachycephalic cats.

Domain *	Low-Grade Severity (Without Clinically Evident UAO)	High-Grade Severity (Clinically Evident UAO)
Owner’s perspective	Presented/admitted for reasons unrelated to UAO.	Presented with UAO-related chief complaints.
Clinical signs	No respiratory clinical signs, or only occasional mild snoring during sleep, or intermittent upper airway noise while awake.	One or more of the following: persistent stertor or stridor at rest or with mild exertion, breathing difficulty after activity, or episodes of respiratory distress attributed to UAO.
Physical examination findings	Stertor/stridor absent, or mild and intermittent, on physical examination and auscultation.	Continuous or frequent loud stertor/stridor on physical examination and auscultation.

* Cats were classified primarily based on patient-level findings. The owner’s perspective domain was considered supportive information, but it was not required for grade assignment. If either the clinical signs domain or the physical examination findings domain met the criteria for high-grade severity, the cat was assigned to the high-grade severity group. Cats were assigned to low-grade severity only when both domains did not meet the high-grade criteria.

**Table 2:** Baseline characteristics of brachycephalic cats with high-grade upper airway obstruction (Brachy-H-UAO), brachycephalic cats with low-grade upper airway obstruction (Brachy-L-UAO), and non-brachycephalic (NB) cats.

Variables	Brachy-H-UAO Cats (*n* = 11)	Brachy-L-UAO Cats (*n* = 7)	NB Cats (*n* = 25)	*p* Value
Age (years)	3.2 (1.0–8.0)	8.0 (5.0–9.0)	2.0 (0.8–6.0)	0.10
BW (kg)	3.6 (±0.9)	4.0 (±0.9)	3.8 (±1.3)	0.75
Female (%)	73	57	48	0.39

Abbreviations: BW, body weight; Brachy-H-UAO, brachycephalic cats with high-grade upper airway obstruction; Brachy-L-UAO, brachycephalic cats with low-grade upper airway obstruction; NB, non-brachycephalic.

**Table 3:** Comparison of BWBP ventilatory variables between brachycephalic cats with high-grade upper airway obstruction (Brachy-H-UAO), brachycephalic cats with low-grade upper airway obstruction (Brachy-L-UAO), and non-brachycephalic (NB) cats.

Variables	Brachy-H-UAO Cats (*n* = 11)	Brachy-L-UAO Cats (*n* = 7)	NB Cats (*n* = 25)	η^2^H	*p* Value
RR (breaths/min)	31 (25–99)	43 (35–58)	62 (50–87)	0.09	0.07
TV/BW (mL/kg)	7.2 (3.5–11.7)	7.2 (4.2–8.8)	8.0 (5.0–10.6)	0.00	0.76
MV/BW (mL/kg/min)	311 (246–386)	253 (233–435)	503 (346–557)	0.21	0.01 *^ab^
PEF/BW (mL/s/kg)	21.9 (18.6–39.9)	13.1 (11.1–17.7)	20.4 (15.3–24.7)	0.25	0.002 *^bc^
PIF/BW (mL/s/kg)	18.8 (14.4–20.7)	16.2 (12.7–25.2)	26.6 (22.4–33.6)	0.26	0.002 *^a^
PEF/PIF	1.46 (1.06–2.37)	0.76 (0.59–0.83)	0.73 (0.64–0.79)	0.50	<0.001 *^ac^
Ti (s)	0.88 (0.27–1.12)	0.55 (0.45–0.63)	0.43 (0.33–0.59)	0.05	0.14
Te (s)	1.18 (0.35–1.57)	0.88 (0.60–1.13)	0.59 (0.48–0.86)	0.02	0.25
Te/Ti	0.74 (0.66–0.94)	0.68 (0.62–0.83)	0.68 (0.62–0.79)	0.01	0.30
PAU	1.50 (0.69–4.45)	0.67 (0.51–0.84)	0.67 (0.54–0.76)	0.20	0.01 *^a^
Penh	2.37 (0.69–11.93)	0.57 (0.34–0.96)	0.53 (0.44–0.61)	0.42	<0.001 *^ac^

Abbreviations: BW, body weight; Brachy-H-UAO, brachycephalic cats with high-grade upper airway obstruction; Brachy-L-UAO, brachycephalic cats with low-grade upper airway obstruction; MV, minute volume; NB, non-brachycephalic; PAU, pause; Penh, enhanced pause; PEF, peak expiratory flow; PIF, peak inspiratory flow; PEF/PIF, peak expiratory flow to peak inspiratory flow ratio; RR, respiratory rate; Te, expiratory time; Ti, inspiratory time; Te/Ti, expiratory time-to-inspiratory time ratio; TV, tidal volume; η^2^H, eta-squared based on the H statistic; significant differences (*p* < 0.05) are denoted by asterisks (*); ^a^, significant difference between Brachy-H-UAO and NB cats; ^b^, significant difference between Brachy-L-UAO and NB cats; ^c^, significant difference between Brachy-H-UAO and Brachy-L-UAO cats.

## Data Availability

The data presented in this study are available upon request from the corresponding author. Part of the data was presented as a poster presentation at the 33rd ECVIM-CA Congress (21–23 September 2023, Barcelona, Spain).

## Supplementary Materials

The following supporting information can be downloaded at: https://www.mdpi.com/article/10.3390/ani16060959/s1, The ARRIVE Essential 10 checklist for the study.
